# Multi-Objective Algorithm for Blood Supply via Unmanned Aerial Vehicles to the Wounded in an Emergency Situation

**DOI:** 10.1371/journal.pone.0155176

**Published:** 2016-05-10

**Authors:** Tingxi Wen, Zhongnan Zhang, Kelvin K. L. Wong

**Affiliations:** 1 Software School, Xiamen University, Xiamen, Fujian, China; 2 School of Medicine, University of Western Sydney, Sydney, New South Wales, Australia; Shenzhen institutes of advanced technology, CHINA

## Abstract

Unmanned aerial vehicle (UAV) has been widely used in many industries. In the medical environment, especially in some emergency situations, UAVs play an important role such as the supply of medicines and blood with speed and efficiency. In this paper, we study the problem of multi-objective blood supply by UAVs in such emergency situations. This is a complex problem that includes maintenance of the supply blood’s temperature model during transportation, the UAVs’ scheduling and routes’ planning in case of multiple sites requesting blood, and limited carrying capacity. Most importantly, we need to study the blood’s temperature change due to the external environment, the heating agent (or refrigerant) and time factor during transportation, and propose an optimal method for calculating the mixing proportion of blood and appendage in different circumstances and delivery conditions. Then, by introducing the idea of transportation appendage into the traditional Capacitated Vehicle Routing Problem (CVRP), this new problem is proposed according to the factors of distance and weight. Algorithmically, we use the combination of decomposition-based multi-objective evolutionary algorithm and local search method to perform a series of experiments on the CVRP public dataset. By comparing our technique with the traditional ones, our algorithm can obtain better optimization results and time performance.

## Introduction

In natural disaster zones where there are serious casualties, it is sometimes necessary to send blood supplies to the injured on their spots instead of bringing them to the hospital for blood infusion due to inaccessibility. With the advancement of unmanned aerial vehicles (UAV) technology (like UAV surveillance[1], traffic monitoring[2], rescue mission[3] and aerial photography[4]) for use in rugged environment, it is easier to substitute UAVs for traditional transportation (such as vehicles, helicopter) in some emergency situations. However, in the event of numerous casualties, a computer program is required to effectively deploy UAVs to their designated locations. This is similar to the travelling salesman problem (TSP) in which the shortest path to be traversed by the salesman to a series of location points is to be determined. However, in real applications, it is much more complicated than the traditional route planning problem, which involves many sub problems such as multiple UAVs’ scheduling, blood’s temperature change during transportation and the different demand of blood in different areas.

The logistics routing problem was firstly proposed by Assad et al. in 1983[5]. The traditional Vehicle Routing Problem (VRP) focuses on how to make the lowest cost when using only one car to send goods from the warehouse to several customers. This problem has been widely studied in many fields, such as network and logistics. In reality, different conditions and situations also bring different constraints to the traditional problem, thus it is extended continuously. For instance, Capacitated VRP (CVRP) [6] limits the vehicle’s carrying capacity, Time-dependent VRP (TDVRP) [7] focuses on the transport time constraints, Multi-depot VRP (MDVRP) [8] introduces multiple starting points into the original problem, and Periodic VRP (PVRP) [9] requires the vehicles’ route planning to be some of time periodicity. At the same time, people are concerned with not only the economic cost in the optimization process, but also the time cost, no-load ratio, etc. For example, in the green logistics [10,11,12,13], people also study the carbon emission problem where the routes in VRP are related to the vehicle emissions.

VRP is a typical NP-hard combinatorial optimization problem [14]. Specifically, when the scale of the problem is not big, the exact algorithm can be used to obtain the optimal solution, such as integer linear programming [15], dynamic programming [16], etc. But with the increment of scale, it is sometimes difficult to solve it in polynomial time. Therefore, we need to use the approximate algorithm to obtain a near-optimal solution. Currently, many researchers use the typical meta-heuristics algorithm [17] to solve the VRP, such as the tabu search [18], simulated annealing [19] in local search method, the bee colony algorithm [20], ant colony algorithm [21] and genetic algorithm [22] in population search method.

Our proposed blood supply problem is an extension to the original CVRP. In the CVRP, there are *n* customers in different locations on the map and each customer has a personal need *d*_*i*_. Also, there are several vehicles which have the same carrying capacity *Q* (*Q* > max{*d*_*i*_| 1≤*i*≤*n*}). We need to arrange and delegate these vehicles to satisfy the need of the injured personnel (our customers). However, each customer can only accept service from one vehicle, each vehicle from the warehouse will finally return without overload during the trip, and the goal of this problem is to minimize vehicles’ mileage. But our problem is to supply the blood to several blood needing places by UAVs which has a limited carrying capacity. However, the blood is transported in the form of blood bags and its temperature will affect its quality, so we need to use the heating agent or refrigerant to keep the temperature in an appropriate range, at the same time, the amount of blood needed or the length of path will affect the weight of the required heating agent or refrigerant. Therefore, the payload of the UAV will be reduced. Because a UAV may need to supply several places in one task, and so the longer the trip, the more heating agent or refrigerant it needs. If we only try to minimize the vehicles’ mileage, it may increase the number of UAVs that is required. In real application, the distance cost is not the only cost in one trip of the UAV, so it is more meaningful to reduce the number of flights. Therefore, we may treat the distance cost and the number of flights as our objectives, which should be optimized in our model. We use the decomposition-based multi-objective evolutionary algorithm to solve this problem and compare it with the traditional multi-objective CVRP algorithm. The experimental result shows that our method can generate a better solution in an acceptance time.

The rest of this paper is organized as follows. In Section 2, we provide a problem definition of the new UAV-based Capacitated Vehicle Routing Problem (UCVRP). Decomposition-based multi-objective evolutionary algorithm with detailed explanation is discussed in Section 3 to solve the new UVCRP. Section 4 gives experimental results. In our experiments, we obtain the relationship among the weight of blood, the heat agent and the route in a certain circumstance, and we also conduct the comparison on the well-acknowledged benchmark instances. The conclusions and further work are drawn in the last section.

## Problem Definition

**Fig 1:**
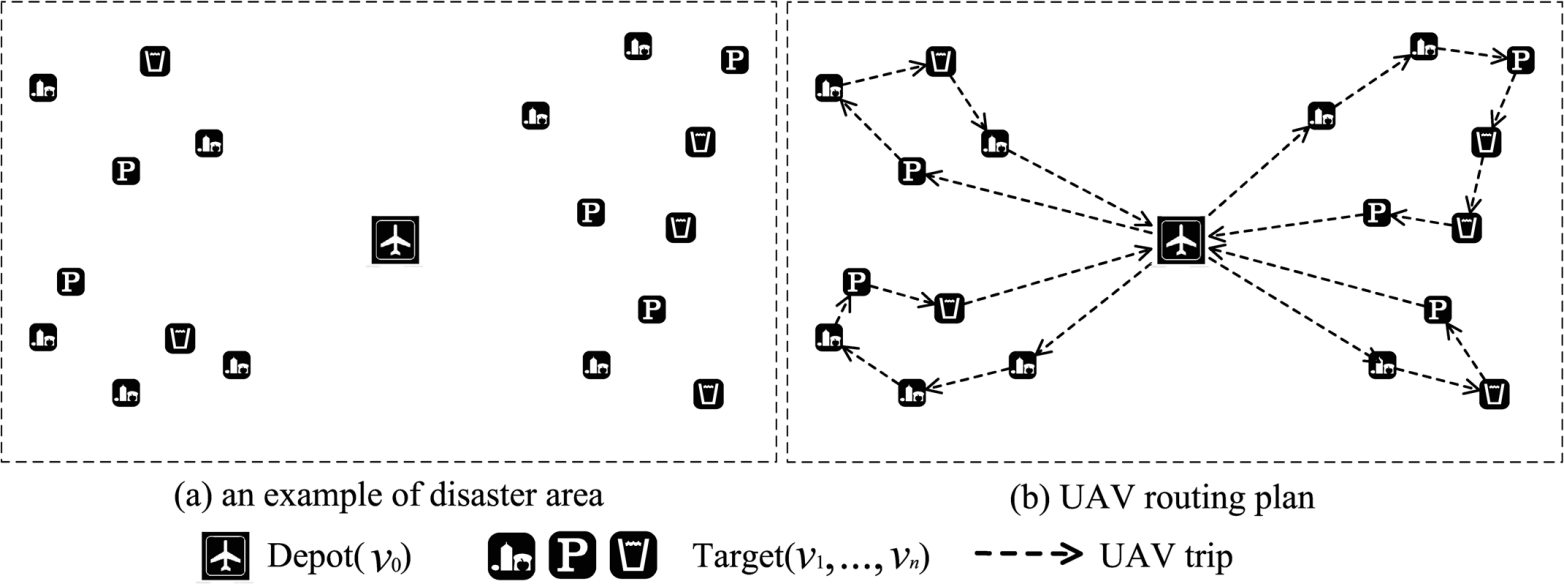
An example of UAV flight plan for multiple disaster areas.

A group of UAVs *U* = {*u*_1_,…,*u*_*m*_} transport the blood bags to each blood needing place from the warehouse. The warehouse (starting point) and several blood needing places forms a bi-directional graph *G* = (*V*,*E*); *V* = {*v*_0_,*v*_1_,…,*v*_*n*_} that consists of the *n* + 1 nodes in the graph and *E* = {(*i*,*j*)|*i*,*j* ∈ *V*,*i* ≠ *j*} is the set of edges; *v*_0_ stands for the starting point while *v*_1_,…, *v*_*n*_ are *n* blood needing places, and *e*_*ij*_ is the flight distance between *v*_*i*_ and *v*_*j*_. Each UAV has a limited carrying capacity *w*, the amount of blood each blood needing place needs is *d*_*i*_, each blood needing place can be supplied at most once, and the UAV will return to the starting point after finishing its task (Fig 1).

During live blood transportation, in order to keep the temperature of the blood within 2–10°C [23], when the environment temperature is below -5°C, some heating agent (hot water) will be placed with the blood bags during the transit. Also, when the environment temperature is higher than 15°C, the refrigerant (ice) will be needed. The hot water and the ice will be called as appendages in the rest of the paper. We hope to have a more reasonable arrangement of the appendages in order to reduce their impact on the payload of UAVs.

In the mathematical model of heat conduction [24], the exothermic process of the hot water is similar to the endothermic process of the ice. For a place *v*_*i*_, the former is defined as follows:

$$-c_{1}•p_{i}\frac{\partial t_{1}}{\partial \tau }=(t_{1}-t_{3})•g_{11}+(t_{1}-t_{2})•g_{31},$$ (1)

$$-c_{2}•d_{i}\frac{\partial t_{2}}{\partial \tau }=(t_{2}-t_{3})•g_{21}+(t_{2}-t_{1})•g_{31}.$$ (2)

The physical meaning of Eq (1) is that the hot water’s releasing heat to the environment and the blood results in the reduction of its enthalpy. Eq (2) denotes the enthalpy reduction of the blood owning to the heat releasing from the blood to the environment and the hot water. The temperature of the blood will rise when the heat from the hot water is greater than the heat flow to the environment; otherwise, it will drop. When the initial temperature of the hot water, *t*_1_(0), the initial temperature of the blood *t*_2_(0) and the temperature of the environment, *t*_3_, are known, we can solve the differential equations. Table 1 summarizes the notations used in the heat conduction model.

**Table 1:** Notations used in the Heat Conduction Model.

Notation	Meaning
*t*_1_	Current temperature of the hot water
*t*_2_	Current temperature of the blood
*t*_3_	Temperature of the environment
*c*_1_	Specific heat capacity of the hot water
*c*_2_	Specific heat capacity of the blood
*g*_11_	Heat conductance between the hot water and the container
*g*_21_	Heat conductance between the blood and the container
*g*_31_	Heat conductance between the container and the environment
*t*_1_(0)	Initial temperature of the hot water
*t*_2_(0)	Initial temperature of the blood
*τ*	Flight time
*p*_*i*_	Weight of the hot water needed for place *v*_*i*_
*d*_*i*_	Weight of the blood needed for place *v*_*i*_

In this model, *c*_1_, *c*_2_, *g*_11_, *g*_21_ and *g*_31_ are constants and relate to the physical characteristics of blood, water and the material of the container respectively. The flight time, *τ*, is determined by the flight distance, *s*_*i*_. Therefore, when *t*_3_, *t*_1_(0) and *t*_2_(0) are given as input values of a specific application environment, the weight of hot water, *p*_*i*_, can be calculated according to Eqs (1) and (2).

Usually, the typical UAV that is used in the emergency situation has a limited flight distance. Therefore, we assume that all the blood needing places are within the reach of UAV and the weight of blood (possible appendages) is not exceeding the carrying capacity. In the UAV scheduling process, *m* UAVs will start from the starting point at the same time and complete the task within one trip. Our goal is to minimize the total mileage and the number of UAVs.

Based on the above model, we can define a complete UAV blood supply route planning problem as follows:

*x*_*ijk*_ is a Boolean variable, *x*_*ijk*_ = 1 indicates that the UAV *u*_*k*_ flies from *v*_*i*_ the *v*_*j*_, otherwise, *x*_*ijk*_ = 0.

*y*_*ik*_ is a Boolean variable, *y*_*ik*_ = 1 indicates that the UAV *u*_*k*_ supplies the *v*_*j*_, otherwise, *y*_*ik*_ = 0.

*S*_*k*_ is a set of nodes visited by the UAV *u*_*k*_.

$$\min Z_{1}=\sum_{i=0}^{n}\sum_{j=0}^{n}\sum_{k=1}^{m}e_{ij}•x_{ijk},$$ (3)

$$\min Z_{2}=m,$$ (4)

$$s.t.\left\{ \begin{array}{l} \sum_{i=1}^{n}(d_{i}+p_{i})\cdot y_{ik}\leq w,\forall k\\ \sum_{k=1}^{m}x_{0jk}\leq m,\forall j\\ \sum_{k=1}^{m}y_{ik}=1,\forall i,i\neq 0\\ \sum_{i=0}^{n}x_{ijk}=y_{jk},\forall j,j\neq 0,i\neq j,\forall k\\ \sum_{j=0}^{n}x_{ijk}=y_{ik},\forall i,i\neq 0,i\neq j,\forall k\\ \sum_{i\in S_{k}}\sum_{j\in S_{k}}x_{ijk}=\left|S_{k}\right|,\forall k\\ S_{k1}\cap S_{k2}=\{0\},\forall k1,\forall k2\\ \underset{k}{\cup }S_{k}=\{0,\ldots ,n\}\\ 0\in S_{k},S_{k}\subseteq \{0,\ldots ,n\},2\leq \left|S_{k}\right|\leq n\\ x_{ijk}=\{0,1\},\forall i,j,k\\ y_{ik}=\{0,1\},\forall i,j,k \end{array}\right..$$ (5)

Objective 1: Eq (3) is used to calculate the total mileage of UAVs, and we minimize the total mileage by obtain the shortest path for each UAV.

Objective 2: Eq (4) is used to obtain the optimal scheduling for minimizing the number of UAVs.

Eq (5) is the constraint for Objective 1 and 2.

Constraint 1: The UAV cannot be overloaded.

Constraint 2: The number of UAVs must be not more than *m*.

Constraint 3: Each blood needing place can only be visited and served by one UAV.

Constraint 4: Each blood needing place is only accessed once.

Constraint 5: Only one UAV departs from each blood needing place.

Constraint 6: The number of edges passed by each UAV is equal to the number of nodes visited by itself.

Constraint 7: Each UAV has to visit the starting point.

Constraint 8: All of the routes of UAVs cover all nodes.

Constraint 3,4,5,6,7,8: All routes should be formed as loops

## Method

**Fig 2:**
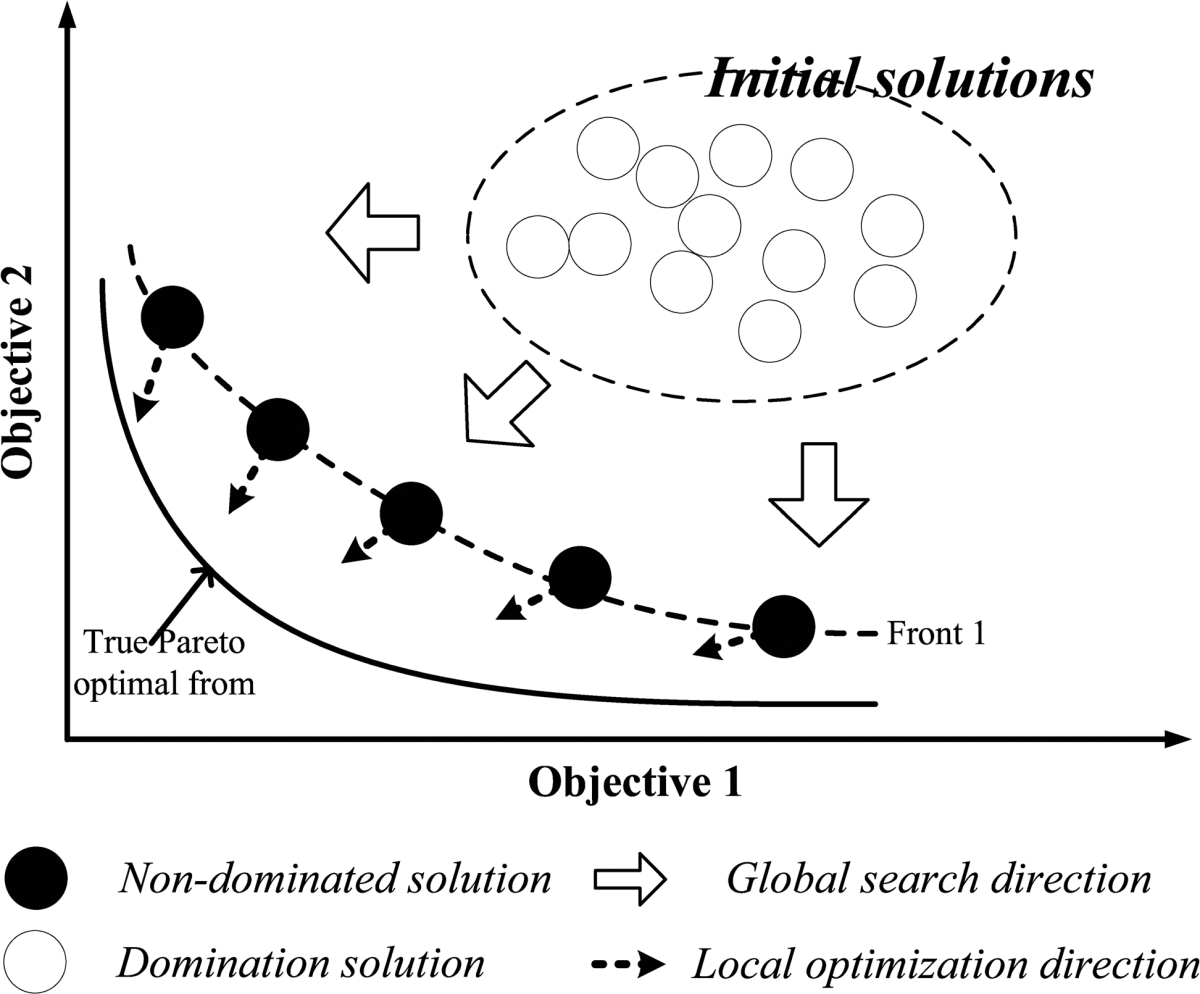
Sketch of multi-objective optimization [26].

UCVRP is a multi-objective problem [25], which attempts to minimize the total distance and the number of required vehicles. The multi-objective optimization is different from the single objective optimization, which will generate a Pareto optimal set having a trade-off between multi objectives. Multi-objective evolutionary algorithms usually initialize a set of random solutions and iteratively generate new ones by selection, crossover, mutation, local search and etc. (as shown in Fig 2)

When dealing with the multi-objective problem, we usually make use of the evolutionary algorithm in two ways. The first is based on the definition of Pareto dominance. By using the non-dominated sort [27], the population will be divided into several levels where the lower level means the better and the non-dominated individuals in the evolution will be reserved by elitist strategy. The second is the decomposition-based multi-objective evolutionary algorithm framework [28]. In this way, the original multi-objective optimization problem will be decomposed into a certain number of single objective sub-problems and each sub-problem is a single objective optimization problem. Thus, the traditional single objective optimization algorithm can be extended and applied in this framework. In this paper, we extend the MOEA/D algorithm framework [28] and use it to solve the UCVRP.

### Chromosome representation

**Fig 3:**
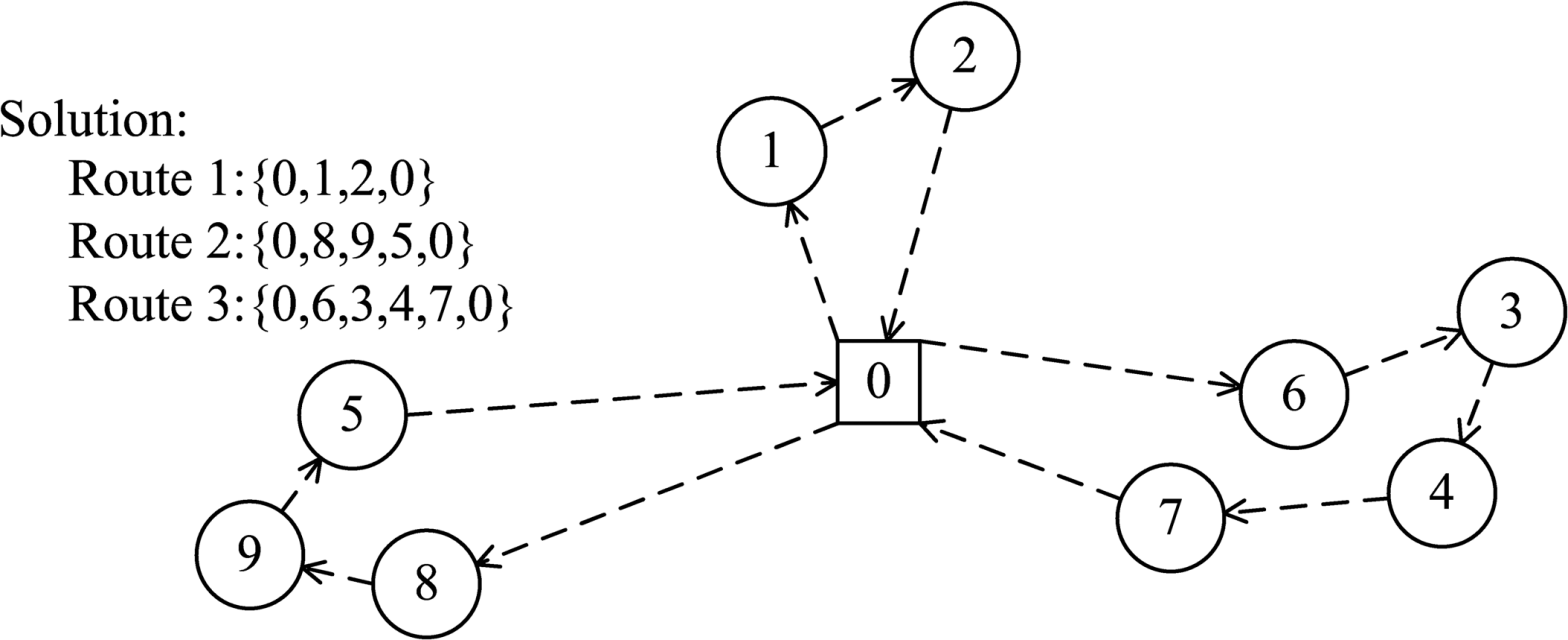
Example of a solution.

Path problem usually uses the natural number coding, that is, using a set of natural numbers *H* = [*h*_0_,*h*_1_,…,*h*_*n*_] to represent the chromosome. There are two common coding methods [29]: the vehicle-oriented natural number coding, and the destination-oriented natural number coding. Fig 3 shows a solution to the UCVRP, where 0 is the starting point of UAVs, number 1–9 represent various blood needing places and the three routes mean that three UAVs are needed to access all the points in one round.

**Vehicle-oriented coding** will firstly determine the number of delivery sub-routes, then place the destination points into sub-routes and determine the point access order for each sub-route (i.e. determine the required number of UAVs, allocate the point required to access for each UAV and determine the order in each sub-route). For example, Table 2 is the vehicle-oriented coding result of the solution in Fig 3, and Target 1 is assigned to Route 1 as the first target to be accessed. The advantage of this method is the decomposition of the problem which reduces the complexity, but it is costly in memory consumption and not intuitive.

**Table 2:** Representation scheme 1: Vehicle-oriented coding.

Target	1	2	3	4	5	6	7	8	9
Route	1	1	3	3	2	3	3	2	2
Sequence	1	2	7	8	5	6	9	3	4

**Destination-oriented coding** directly uses a one-dimensional array to represent the route. For instance, Table 3 is the destination-oriented coding result of the solution in Fig 3. Although this method can clearly obtain the optimized route and is easily to be implemented, it often generates many infeasible solutions and is not conducive to the optimization of the solution.

**Table 3:** Representation scheme 2: Destination-oriented coding.

0	1	2	0	8	9	5	0	6	3	4	7	0

These two methods are commonly used when the number of vehicles or the number of routes is known. Because the total number of vehicles is one of our optimizing goals in this paper, therefore, we choose the destination-oriented coding and eliminate all 0s from it. By partitioning the route from left to right, we can get gene segment which is not exceeding the carrying capacity as a route (as shown in Table 4). This representation of the solution uses less computer memory and is more intuitive; moreover, the solution generated by this method must be a feasible one.

**Table 4:** Representation scheme 3.

Route1		Route2			Route3			
1	2	8	9	5	6	3	4	7

### Crossover

**Fig 4:**
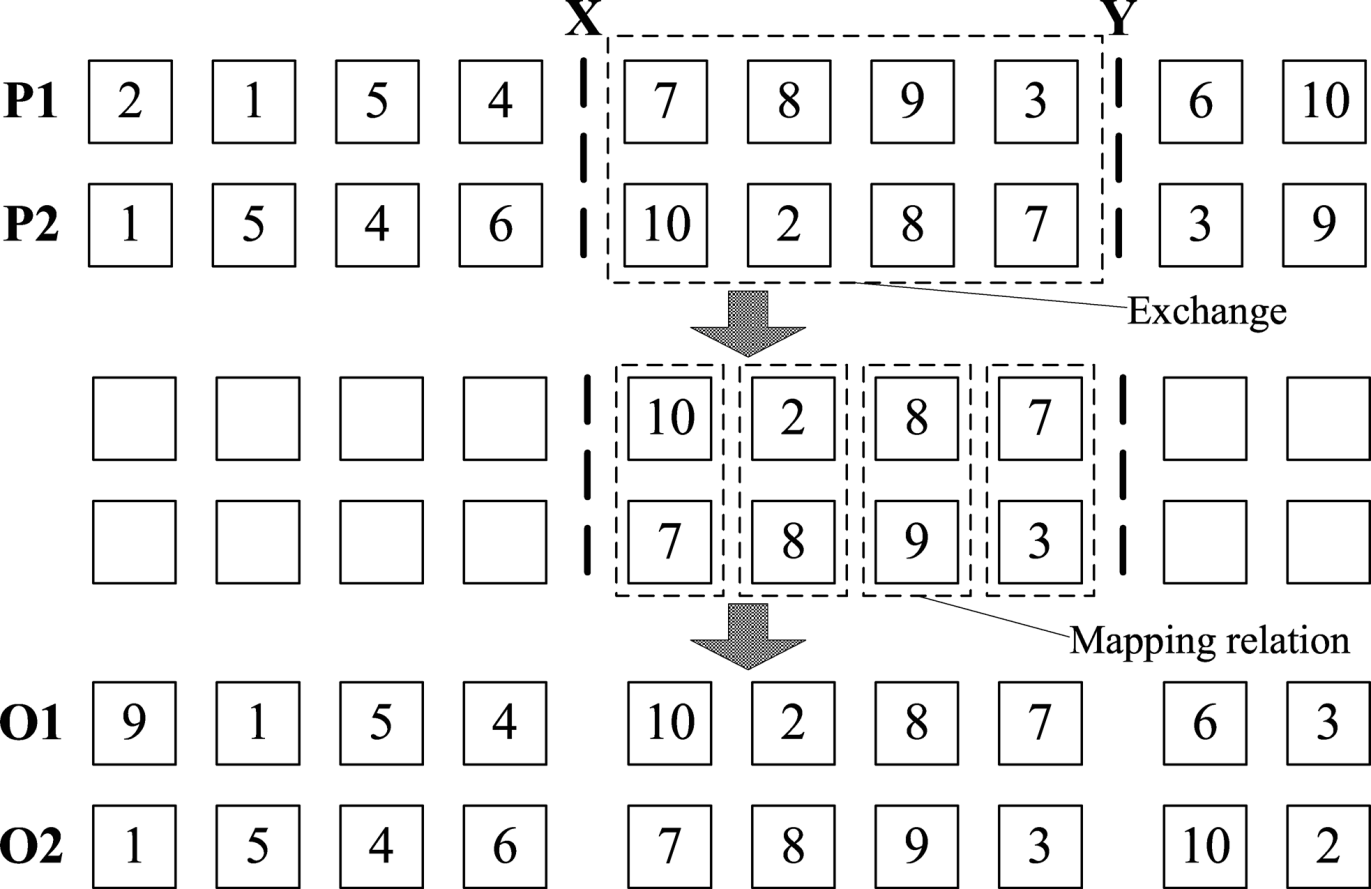
Example of PMX operation.

In genetic algorithm, the crossover can generate new individuals by swapping the partial structure of two parents and we can use it to greatly improve the searching ability of the genetic algorithm. For the permutation encoding, there are some common crossover algorithms, such as Partially Matched Crossover (PMX), Position-based Crossover, Order Crossover, Cycle Crossover [30]. In this paper, we use the classical crossover algorithm PMX. Since the accessed points are placed in chromosome with the order from left to right, so if we try to directly swap the partial structure of two parents, there may exist some points which appear twice or disappear in children, and such chromosomes will not meet the constraints of the model. Firstly, PMX will randomly select two cutting points X and Y from the two parents, swap the segment between X and Y from one parent with that from the other parent and record the mapping relation for this swap. For the remaining part of each parent, it’s by using the relation that we map the old value to a new value. (As shown in Fig 4)

### Mutation

**Fig 5:**
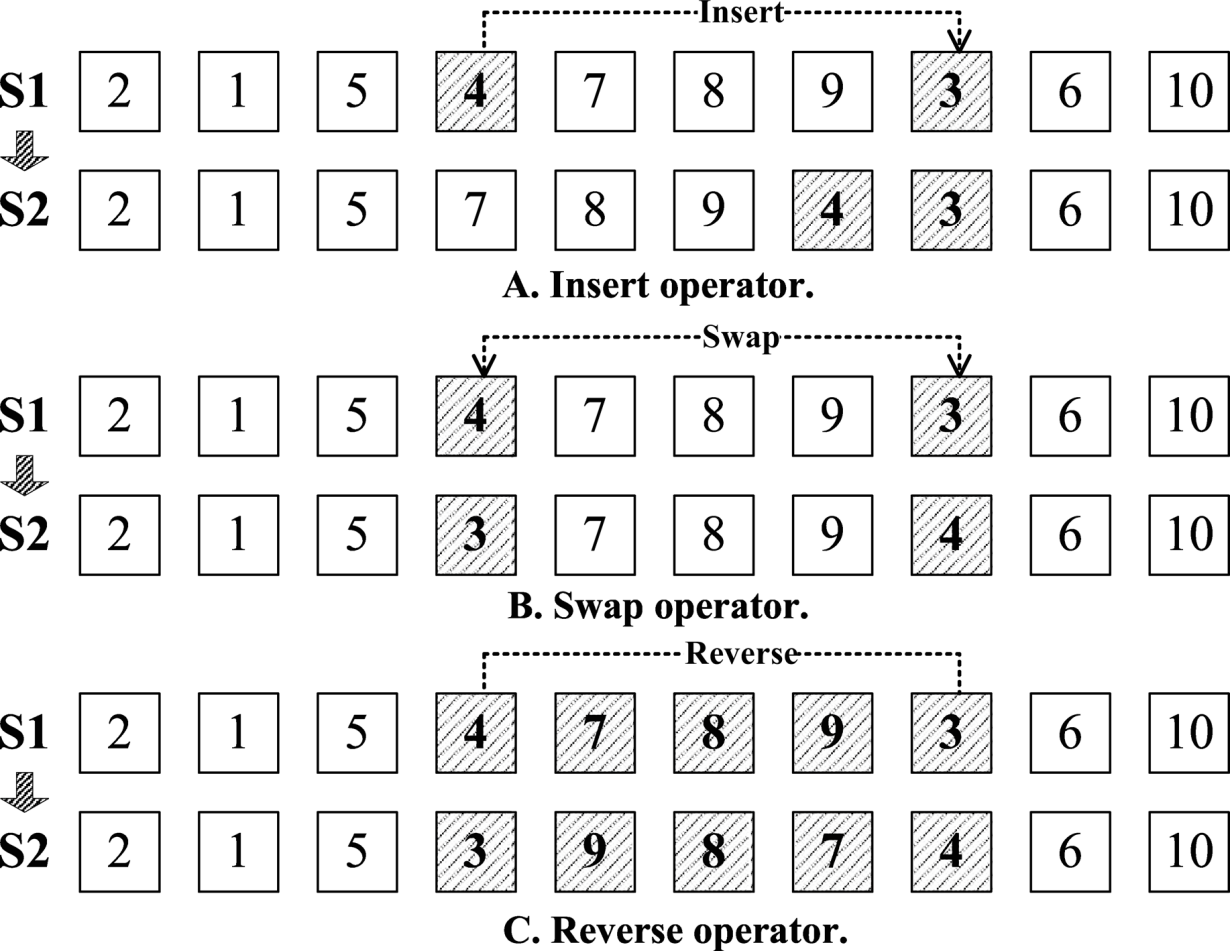
Mutation operators.

Mutation is usually used in genetic algorithm to increase the diversity of the population. When dealing with the route encoding, in order to avoid the crossing, we use the gene segment to represent the route and do the insertion, swap and reverse to optimize it by various ways [31,32]. (As shown in Fig 5)

### Local search

Although the genetic algorithm is a global optimization algorithm, its local search ability is poor since its local search ability is mainly realized by mutation, which is more suitable for searching in a large scale. Therefore, it has a poor performance in the local search, in another word, the fine-tune ability of the mutation is limited. In this paper, the local search heuristics is introduced to improve the local search ability of this algorithm.

In the Holland’s schema theorem [33], the representation and the reproduction of the artificial chromosome has been qualitatively analyzed and discussed. Since the schema can also be interpreted as the same structure, it can represent the structure which has the same features in the chromosome. Based on the encoding method in this paper, the schema is a route or a sub-route, and we can get the expectation of the number of individuals which contains the schema S in generation t+1 according to the HOLLAND schema theorem,

$$n_{t+1}(S)\geq n_{t}(S)\times \frac{f(S)}{f_{avg}}\times \left[1-P_{c}\frac{\delta (S)}{l-1}-O(S)P_{m}\right],$$ (6)

where *n*_*t*_(*S*) and the *n*_*t*+1_(*S*) represents the number of individuals, which contains the schema *S* in generation *t* and *t*+*1* respectively; *f*(*S*) is the average fitness of individuals contains the schema, while the *f*_*avg*_ is the average fitness of the whole population; *δ*(*S*) is the defined length of the schema; *l* is the length of each chromosome; the probability of the crossover and the mutation is *P*_*c*_ and *P*_*m*_ respectively; *O*(*S*) is the number of definite characters in the schema. The determinants in the number of individuals contains the schema in generation *t*+1 are described in the definition of the schema theorem, which mainly includes the ratio of the average fitness of individuals contains the schema to the average fitness of the population, probability of the crossover and the probability of the mutation. It can be seen that after the operation of selection, crossover and mutation in genetic algorithm, the appearance possibility of low-order schemata, shorter defining length and higher average fitness will increase exponentially. When we use the random connection strategy that nearest-neighbors have the priority, the generated schema and its sub-schema usually have the segment of the optimal solution with low-order schemata, shorter defining length and higher average fitness and these segments are easy to be passed to the next generation.

**Fig 6:**
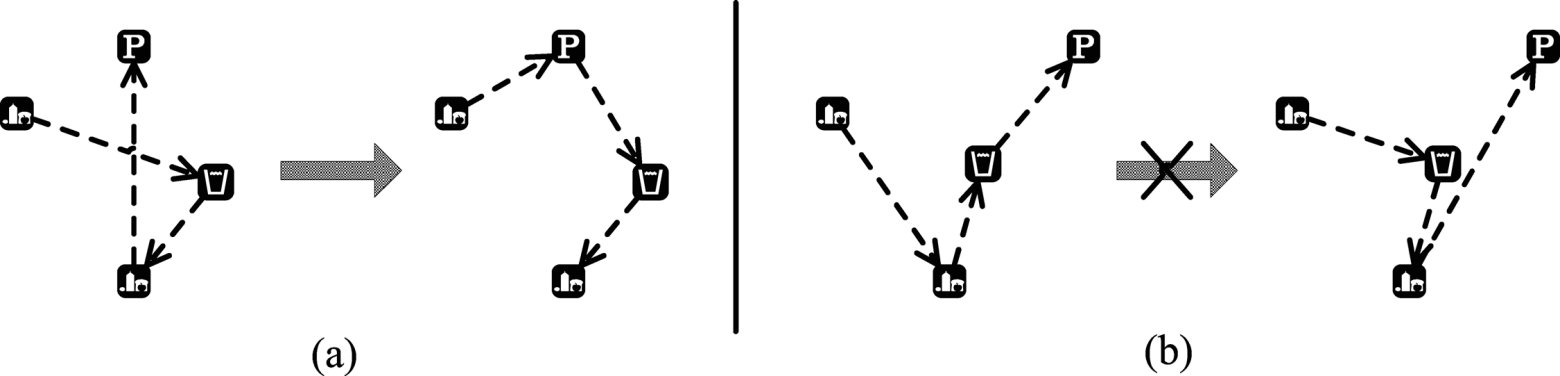
Results of nearest neighbor reorganization.

The basic idea of the strategy is that we divide the code sequence of one chromosome into several segments from which some segments are randomly selected at a proper proportion. For each selected segment, we select the first element as the first one for the new segment after reorganization, select the nearest element to the first one from the remaining elements of the original segment as the second one for the new segment, and then select the nearest one to the second element as the third one, until all elements from the original selected segment have been processed. The fitness of the recombined gene code will be calculated and compared with the original one. If the new recombined gene code is dominated by the original one, the new one will be discarded; otherwise the new one will replace the old one. As shown in Fig 6(A), the new segment has a better fitness and will replace the old one. However in Fig 6(B), the old one is better and the new one is discarded.

Because the algorithm needs to repeatedly use the order of distances between all nodes, in order to avoid repeatedly calculating the distances and the orders, we build the adjacency matrix *R*. Assuming there is a *N*×*N* distance matrix *D*, *D*_*ij*_ represents the distance between nodes *i* and *j*. *R*_*ij*_ is the order of distance *D*_*ij*_ among all the distances *D*_*ik*_ (*k*∈[0,*N*-1], *k*≠*i*). For example, the *R*_02_ = 1 represents that node 2 is the nearest to node 0 and the *R*_01_ = 2 represents that node 1 is the second nearest to node 0.

$$D=\left[\begin{array}{ccc} 0 & 6.22 & 5.51\\ 4.70 & 0 & 6.34\\ 8.53 & 5.72 & 0 \end{array}\right],\;R=\left[\begin{array}{ccc} 0 & 2 & 1\\ 1 & 0 & 2\\ 2 & 1 & 0 \end{array}\right].$$

The detail process of the random nearest neighbor search is as follows:

Step 1: Evenly divide the gene code into βM segments (M is the number of UAVs required in this solution while β is used to control the segment size).

Step 2: Select β*M*θ gene segments according to the recombination proportion θ.

Step 3: According to the adjacency matrix R, the selected gene segments are reorganized based on the random nearest neighbor connection strategy.

Step 4: Complete the recombination of β*M*θ gene segments and generate the new gene code.

### Algorithm framework

**Fig 7:**
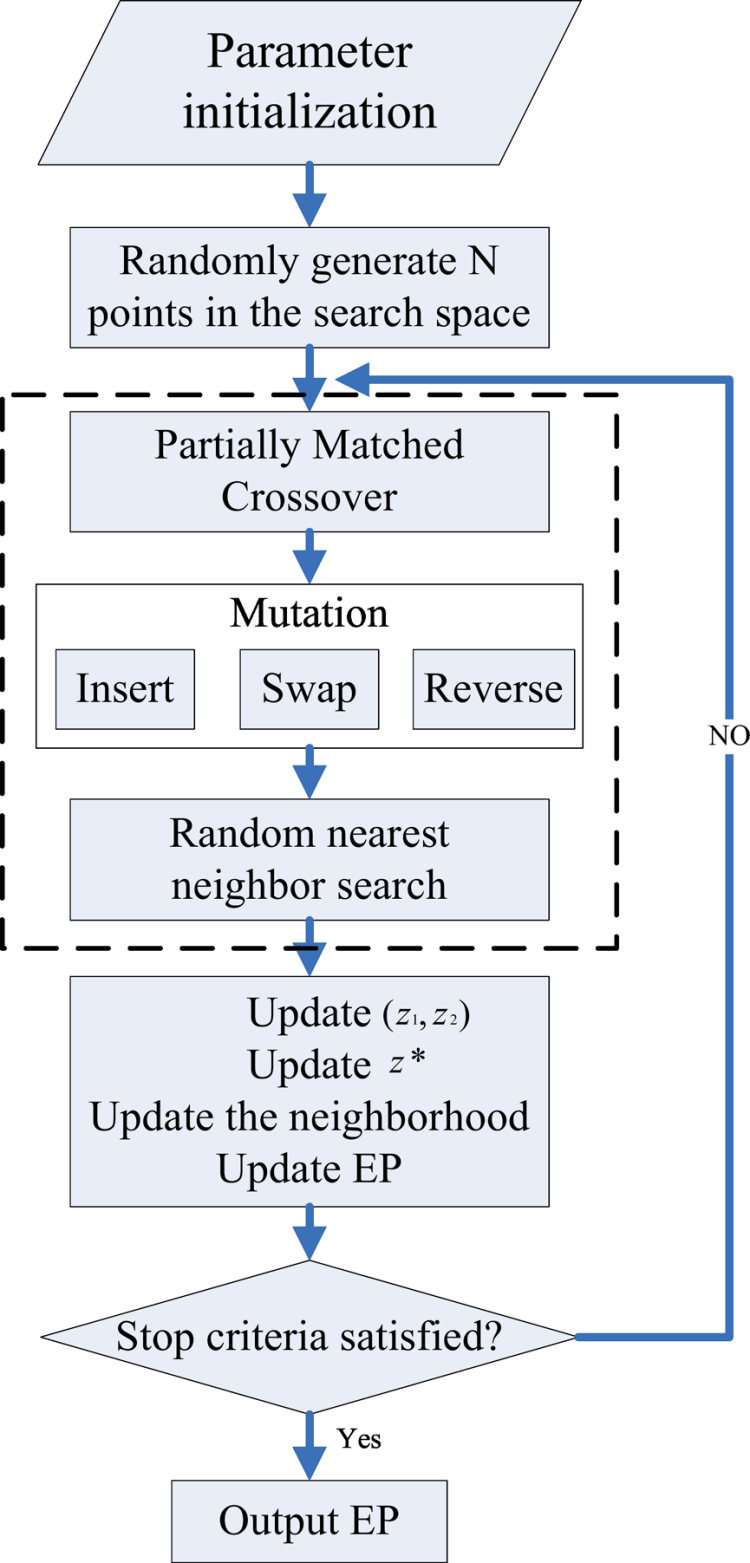
MOEA/D-N-UVRP that is based on the framework of the MOEA/D for UCVRP, where the main adjustment is in the dashed box.

In this paper, we extend the framework of decomposition-based MOEA and apply the local search with random nearest neighbor to solve the UCVRP. The framework of our proposed algorithm, MOEA/D-N-UVRP, is shown in Fig 7.

Besides, we also use two other algorithms, MOEA/D [28] and NASGII [27], in order to solve the UCVRP for comparison. Here, we name them as MOEA/D-UCVRP and NASGII-UCVRP respectively. All three algorithms adopt the strategy of mutation and crossover; however, the random nearest neighbor search is our contribution.

## Experimental Result

### Calculation of the proportion between blood and appendage

**Fig 8:**
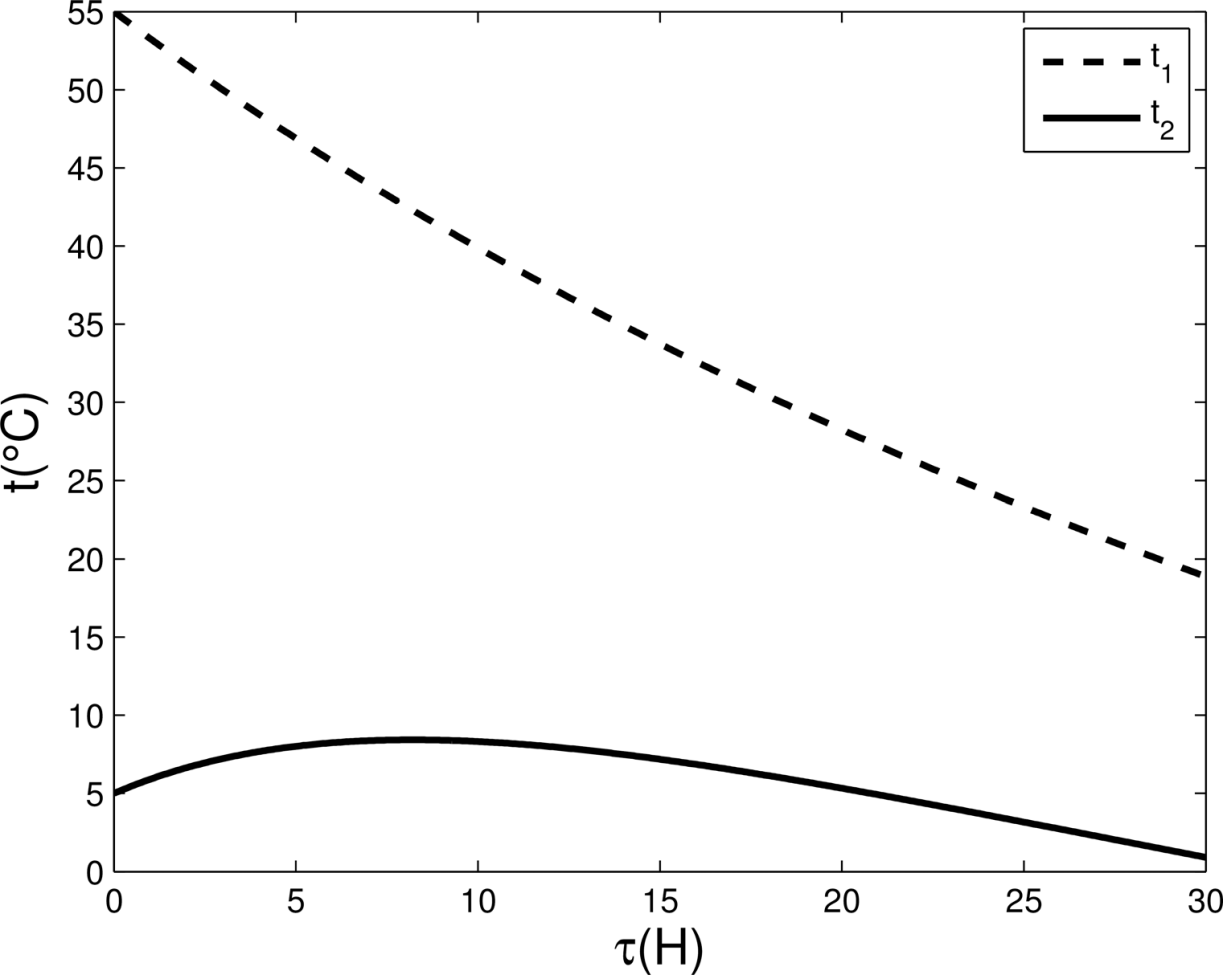
The temperature of the water (*T*_*1*_) and the blood (*T*_*2*_) change with time in the given circumstance.

In our model, the UAV carries blood and appendage, and we can calculate the weight of required appendage by using the weight of blood required and the transport time. Assuming that the temperature of the external environment is -25°C, 2000ml blood that has an initial temperature, *T*_2_(0) = 5 based on 2.4 kg of hot water with an initial temperature, *T*_1_(0) = 50, and that the current temperature of the blood transportation container is 20°C. According to the Eqs (1) and (2), T_1_ and T_2_ will change as shown in Fig 8.

**Fig 9:**
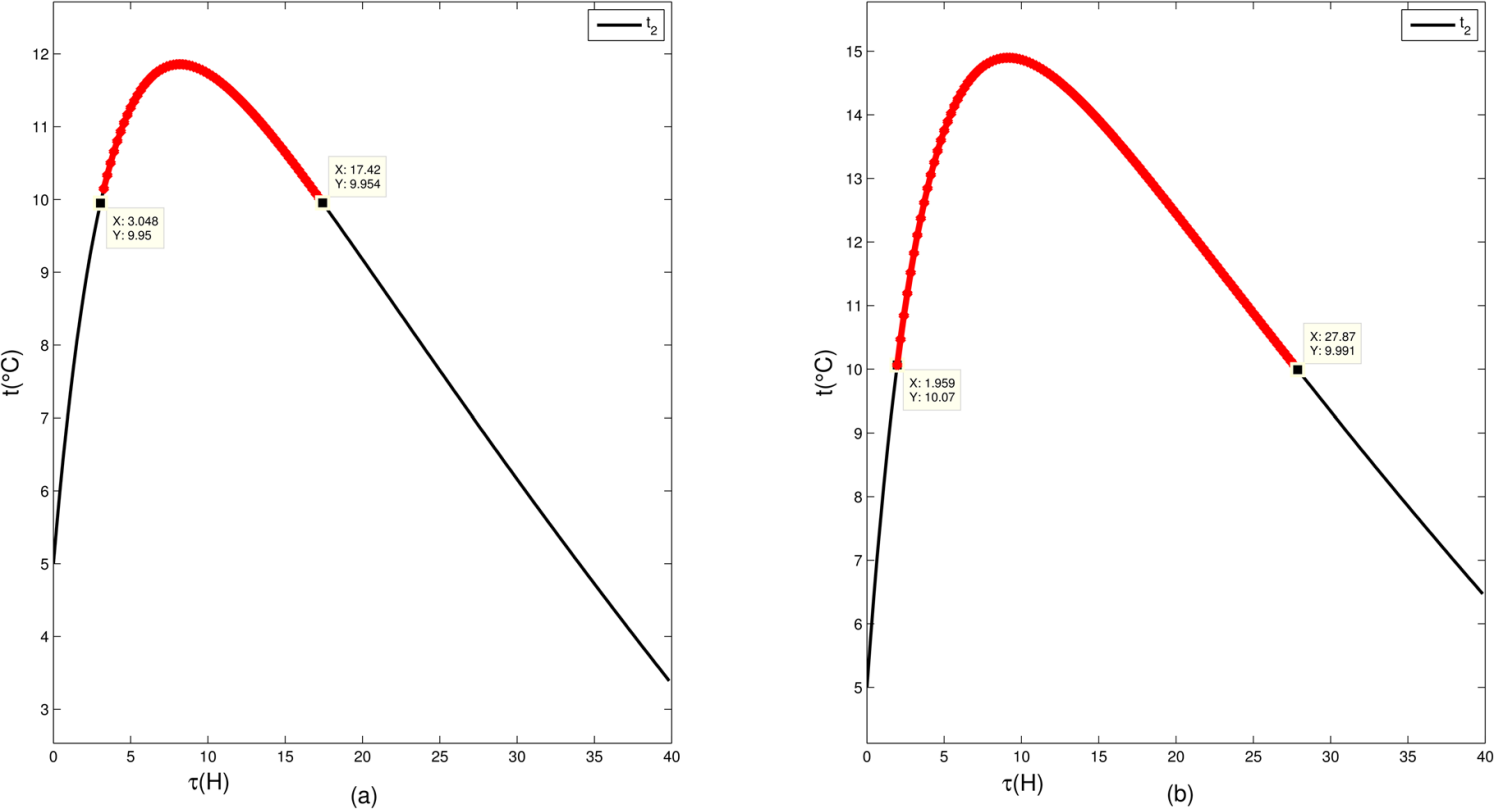
Temperature changes with time in the case of different weight of hot water and blood. Note: Red line represents the blood temperature is beyond the appropriate range.

It can be seen from Fig 8 that the temperature of the water is decreasing with time whereas the temperature of the blood is increasing during the time as the water temperature is decreasing. In the problem, we must ensure that the temperature of the blood is in an appropriate range during the transit. If the weight of the hot water is relatively heavier than the weight of the blood, the blood temperature will rise rapidly in a short period of time. In Fig 9(A), when the ratio of the weight of hot water to the weight of blood is 4.4, the blood temperature can be kept between 2–10°C (in the appropriate range) within 4.5 hours, and above 10°C between 4.5 hours and 12.6 hours. Then in Fig 9(B), we increase the initial temperature of hot water, the blood temperature will be above 10°C within just 2 hours and last for more than 20 hours. Therefore, during the transit, the change of the blood temperature depends on the initial temperature and the weight of hot water, and it’s not practical to increase the initial temperature of the hot water for the goal of reducing its total weight.

**Fig 10:**
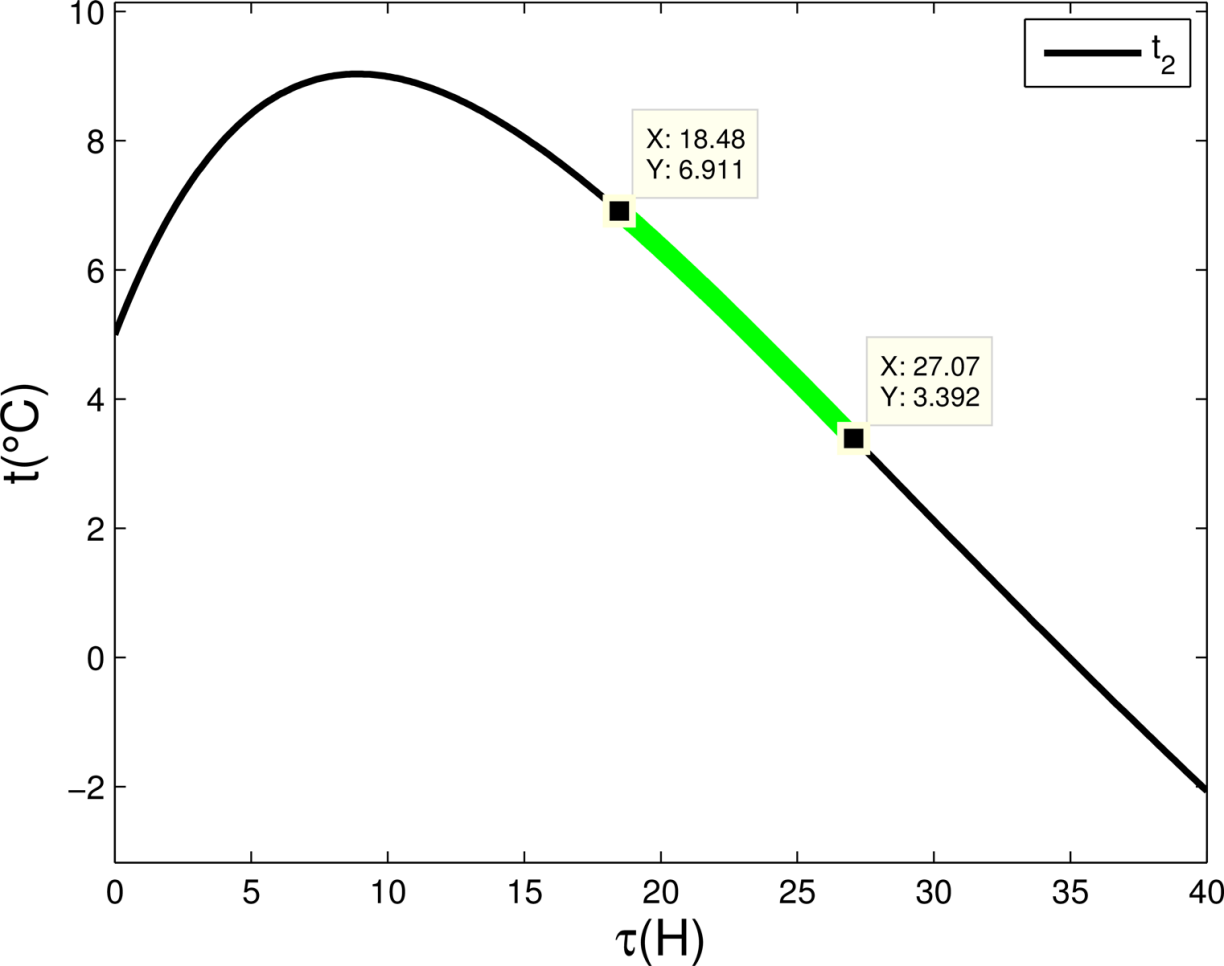
Temperature changes with time. Green line represents an appropriate time interval of arrival.

In our real application, in addition to keeping the blood temperature within the appropriate range, considering that the blood may not be used immediately, the blood should have a proper temperature in a longer period of time. As shown in Fig 10, in order to use less hot water while keeping the temperature of the blood in an appropriate range after delivery, we can choose the delivery time when the temperature in the descending zone.

In the optimization of the UAVs’ scheduling, the evolutionary algorithm requires a lot of iterations, and it will cost a computational time to calculate the weight of required hot water per iteration. In order to reduce the calculation time, we draw a hot water weight proportion table according to the blood weight and transit time in the real situation at a given temperature as shown in Table 5 (The horizontal axis shows weight and the vertical axis shows distance). For example, when the flight distance to one place is 7 and the weight of blood is 11, the weight of hot water needed is 11×0.06.

**Table 5:** Proportions between hot water and blood.

Distance/Weight	[0, 2]	[2, 3]	[3, 5]	[6, 10]	[10, 15]	[15, 20]
[0, 5]	1	0.5	0.2	0.06	0.03	0.02
[5, 10]	1.2	0.8	0.4	0.10	0.06	0.04
[10, 15]	1.9	1.2	0.6	0.18	0.12	0.08
[15, 20]	2.4	1.5	0.8	0.28	0.18	0.15

Since the experiment is based on the VRP public data set, the data set has only the numerical values without unit and its distribution tends to be discrete. So when calculating the weight of required hot water, we map the weight of blood needed and the distance to the range [0, 20] according to Eq 7, where the distance is linear with the time, which can be mapped directly into the time interval. We also note that Eq 7 is a linear transformation.

$$\begin{array}{l} coordX=20•\frac{CurX-MinX}{MaxX-MinX}\\ coordY=20•\frac{CurY-MinY}{MaxY-MinY} \end{array}.$$ (7)

In that equation, *CoordX* is the mapped value of the blood’s weight, *CoordY* is the mapped value of the distance. Now, *MaxX* and *MinX* are maximum and minimum loadage respectively, while *MaxY* and *MinY* are maximum and minimum flight distance respectively. However, since the weight of blood and hot water may exceed the carrying capacity of the UAV during transit, there may exist some nodes which cannot be accessed in the data set.

In the experimental process, the starting point also can be considered as a destination point, since the blood requirement and the flight distance is zero, so we have *MinY* = *MinX* = 0 here. When mapping the blood weight, if the weight of blood is close to the *MaxX*, the minimum ratio of the weight of hot water to the weight of blood can be set as 0.02:1. In order to ensure that the weight of blood and hot water will not exceed the maximum carrying capacity of the UAV, the *MaxX* can be set as *w* / (1 + 0.02). As for the mapped value of distance *MinY*, we can set it directly for different data instances.

**Fig 11:**
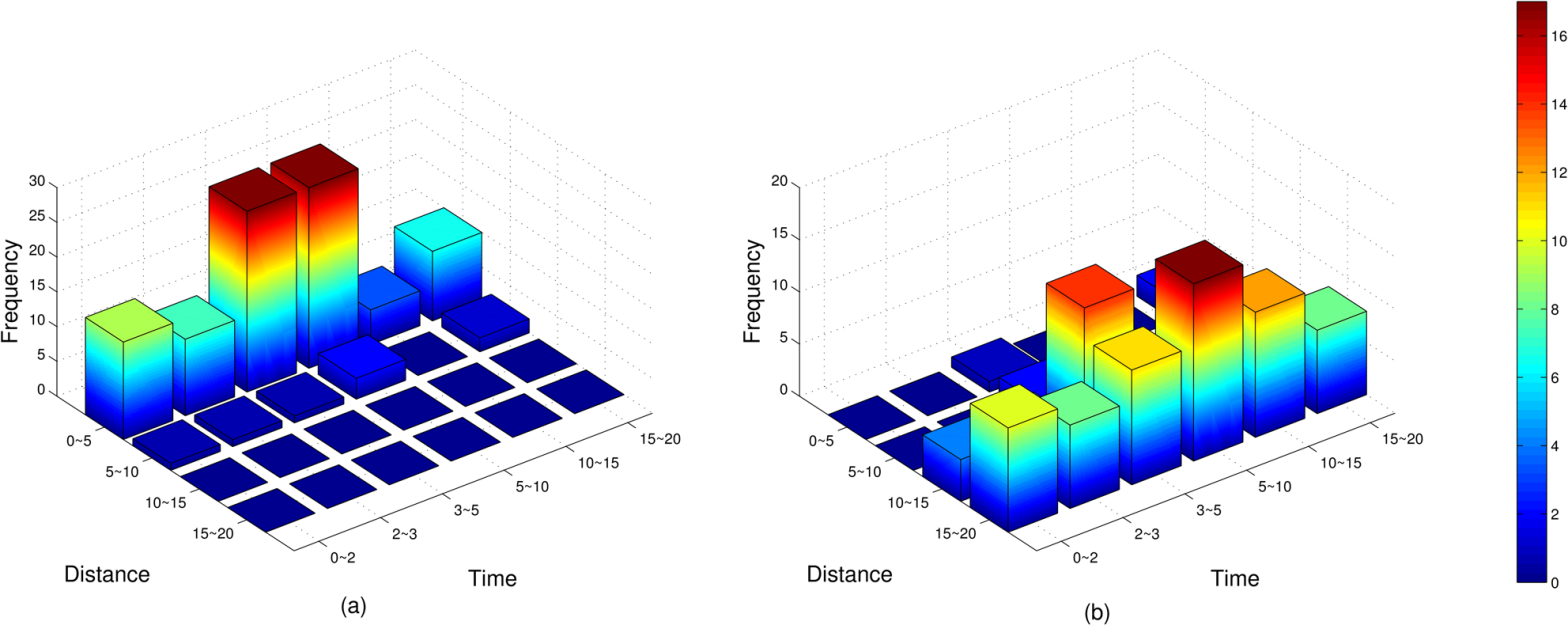
Using frequency of each proportion in Table 5 for instance E-n101-k14 according to different *MaxY* values. *MaxY* is set as 150 and 30 for (a) and (b) respectively.

It can be seen from Table 5 that the change of the proportion is not uniform; the proportion value increases along the time axis from small to large and decreases with the blood weight. While the *MinX* for blood weight has been given above, the *MaxY* for distance is not set yet. As shown in Fig 11, when the *MaxY* takes a large value, the proportion between hot water and blood is easy to be concentrated in the smaller area on the time axis; however, if the *MaxY* takes a small value, the proportion is easy to be concentrated in the larger area. Fig 11 shows the distribution of the proportion caused by different *MaxY* values. If the *MaxY* takes a larger value, it will result in a larger requirement of the hot water, then the total weight UAVs need to carry will increase and the more UAVs will be needed.

### Parameter setting

Parameters used in the experiments are shown in Table 6. All the algorithms are written using Java programming language and operate on a computer (Core I3 CPU, 2.93GHZ, 4G memory space). All the operating computers adopt single-threaded execution.

**Table 6:** Algorithm parameters setting.

Parameter	Value
Population size	100
Crossover rate	0.95
Mutation rate	0.2
Iteration number	15000000
Local search rate, β value, recombination ratio	0.3, 1~3, 0.5

### Two-objective: total distance and number of UAVs

In traditional CVRP, the total distance and the number of vehicles cannot be the two partially conflicting objectives. If the total distance is the minimal one, we cannot reduce it by adding additional vehicles. In our problem, we should consider increasing the payload and reducing the carry of the hot water. If the number of UAVs is small, then the UAVs will need to fly a longer time. However, that means we need more hot water. Thus, the total distance and number of UAVs are both relevant and competitive.

**Fig 12:**
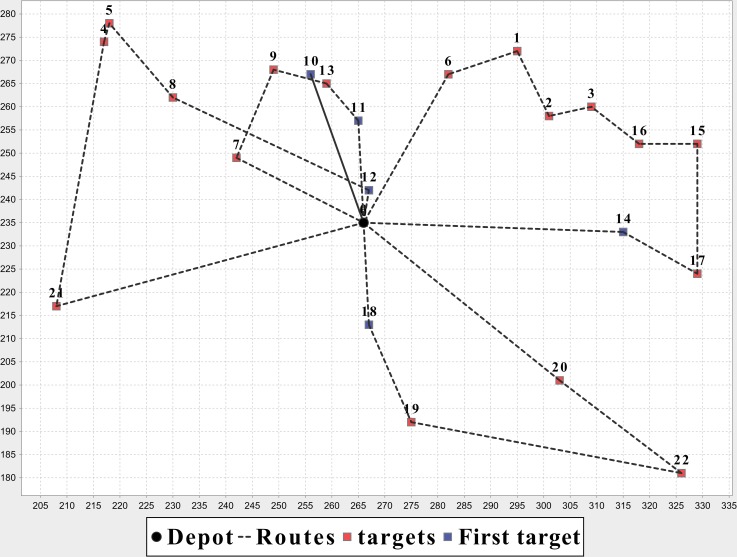
Optimization results of MOEA/D-N-UAV on instance E-n23-k3.

**Fig 13:**
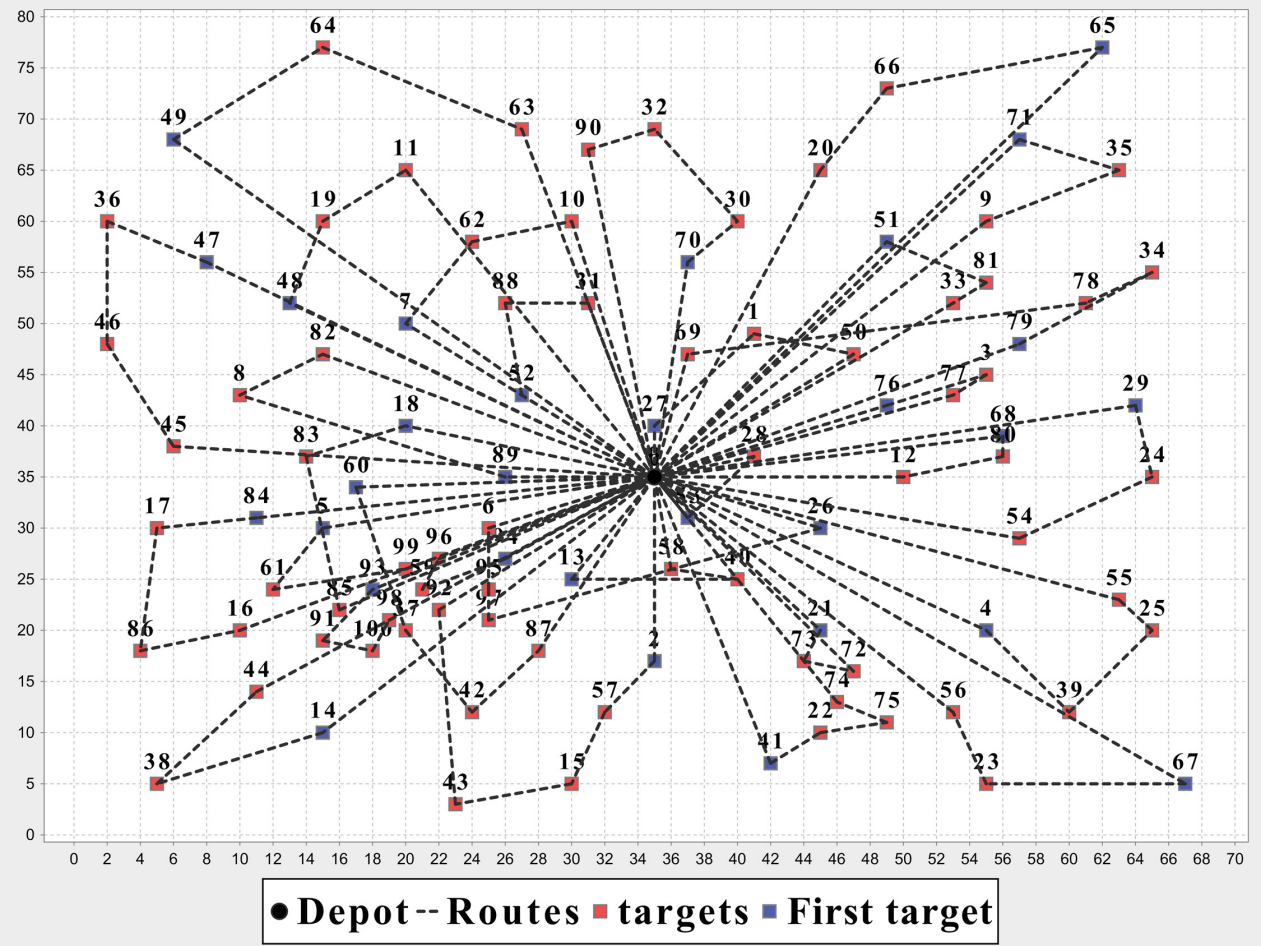
Optimization results of MOEA/D-N-UAV on instance E-n101-k14.

In Table 7, the MOEA/D-N-UAV is the algorithm proposed in this paper for UCVRP, the NSGAII-CUVRP is based on the traditional NSGAII for CUVRP and the MOEA/D-CUVRP is based on the MOEA/D. We run these three algorithms on nine instances according to different proportions between hot water and blood. Each algorithm has been executed five times and we take the average of the results. It’s shown that the MOEA/D-N-UAV is better than the MOEA/D-CVRP and the NSGAII-CVRP in terms of optimization, and has an average increase of 3% compared with MOEA/D-CVRP. The detail optimal solutions of MOEA/D-N-UAV on instance E-n23-k3 and E-n101-k14 are illustrated in Table 8, Figs 12 and 13.

**Table 7:** Computational results of numerical experiments.

Instance	*MinY*	MOEA/D-N-UVRP		NSGAII-CUVRP		MOEA/D-CUVRP	
		Routes	Distance	Routes	Distance	Routes	Distance
E-n23-k3	50	5	716.0254	5	796.1452	5	796.2122
	100	5	720.3495	5	729.5467	5	809.4691
E-n30-k3	50	7	774.9813	7	796.4517	7	821.2371
	100	7	766.8213	7	830.3797	7	803.5596
E-n22-k4	50	7	562.3227	7	562.8716	7	568.0511
	100	7	552.6351	7	553.9392	7	591.9198
E-n30-k4	50	7	555.9675	7	555.9675	7	564.2600
	100	7	555.8100	7	559.5431	7	571.6969
E-n33-k4	50	7	563.0726	7	584.1540	7	564.6380
	100	7	553.9392	7	557.3958	7	570.1265
E-n51-k5	50	14	929.4797	14	945.5339	14	977.78299
	100	13	931.3218	14	933.0224	13	937.06053
E-n76-k7	50	19	1594.2543	20	1337.2121	20	1362.9699
		20	1326.8182				
	100	19	1359.2679	19	1500.9811	19	1461.3264
		20	1347.6392			20	1401.1281
E-n76-k14	50	26	1599.4121	26	1663.6542	26	1640.9537
	100	26	1593.0755	26	1597.5972	26	1640.9537
E-n101-k14	50	30	1942.2819	30	1942.2819	30	1992.8213
	100	30	1929.004	30	1980.2141	30	2026.7598

**Table 8:** Detailed optimization results of MOEA/D-N-UAV on instance E-n23-k3 and E-n101-k14.

Instance	*MinY*	Distance	Routes	Optimal solution
E-n23-k3	50	716.5428	5	Route #1: {0, 11, 13, 9, 7, 0}
				Route #2: {0, 18, 19, 22, 20, 0}
				Route #3: {0, 14, 17, 15, 16, 3, 2, 1, 6, 0}
				Route #4:{0, 12, 8, 5, 4, 21, 0}
				Route #5:{0, 10, 0}
E-n101-k14	50	1938.8909	30	Route #1: {0, 71, 35, 9, 0}
				Route #2: {0, 65, 66, 20, 0}
				Route #3: {0, 68, 80, 12, 0}
				Route #4: {0, 27, 1, 50, 0}
				Route #5: {0, 47, 36, 46, 45, 0}
				Route #6: {0, 48, 19, 11, 0}
				Route #7: {0, 79, 34, 78, 69, 0}
				Route #8: {0, 89, 8, 82, 0}
				Route #9: {0, 26, 97, 95, 6, 0}
				Route #10: {0, 51, 81, 33, 0}
				Route #11: {0, 60, 37, 42, 87, 0}
				Route #12: {0, 18, 83, 85, 0}
				Route #13: {0, 7, 62, 10, 0}
				Route #14: {0, 94, 59, 96, 0}
				Route #15: {0, 53, 28, 0}
				Route #16: {0, 13, 40, 58, 0}
				Route #17: {0, 70, 30, 32, 90, 0}
				Route #18: {0, 93, 91, 100, 98, 0}
				Route #19: {0, 76, 3, 77, 0}
				Route #20: {0, 41, 22, 75, 74, 0}
				Route #21: {0, 14, 38, 44, 0}
				Route #22: {0, 5, 61, 99, 0}
				Route #23: {0, 4, 39, 25, 55, 0}
				Route #24: {0, 29, 24, 54, 0}
				Route #25: {0, 67, 23, 56, 0}
				Route #26: {0, 21, 73, 72, 0}
				Route #27: {0, 49, 64, 63, 0}
				Route #28: {0, 84, 17, 86, 16, 0}
				Route #29: {0, 2, 57, 15, 43, 92, 0}
				Route #30: {0, 52, 88, 31, 0}

In the actual experiments, although the total distance and the number of UAVs show some competition, they are not completely antagonistic. As shown in Table 7, for instance E-n76-k7, the MOEA/D-N-UCVRP can obtain two optimal solutions while the NSGAII-CUVRP only has one. From the results of MOEA/D-N- UCVRP, the total distance by 20 UAVs is less than the one by 19 UAVs. Also, we can adjust the *MinY* to generate more solutions which indicates that introducing the concept of the appendage into UCVRP makes distance and weight be associated and competitive so that generate the UCVRP.

### Performance analysis

The main metrics of the multi-objective algorithm are Hypervolume (HV) and Inverted Generational distance (IGD) [34]. HV computes the size of the region that is dominated by a set of non-dominated solutions, based on a reference vector that is constructed using the worst objective values of each objective. It is defined as

$$HV(PF)=U_{f\in PF}HV(f)\;\text{with}\;HV(f)=\{f^{\prime }\in O:f≺f^{\prime }\},$$ (8)

where the Pareto front (PF) denotes the set of non-dominated sets, *O* is the objective space and HV(*f*) is the set of objective vectors dominated by f. Distance from Representatives in the PF (IGD-metric): Let P* be a set of uniformly distributed points along the PF. Let *A* be an approximation to the PF, the average distance from *A* to P* is defined as:

$$D(A,P*)=\frac{\sum_{v\in P*}d(v,A)}{\left|A\right|},$$ (9)

where *d*(*v*, *A*) is the minimum Euclidean distance between v and the points in *A*.

However, in our model, the total distance and the total number of UAVs are not completely antagonistic. The optimization results of these three algorithms only have little non-dominated solutions each time, usually, the number is 1 to 2. Then the PF may have only one solution, which cannot be calculated by Eqs (8) and (9).

**Fig 14:**
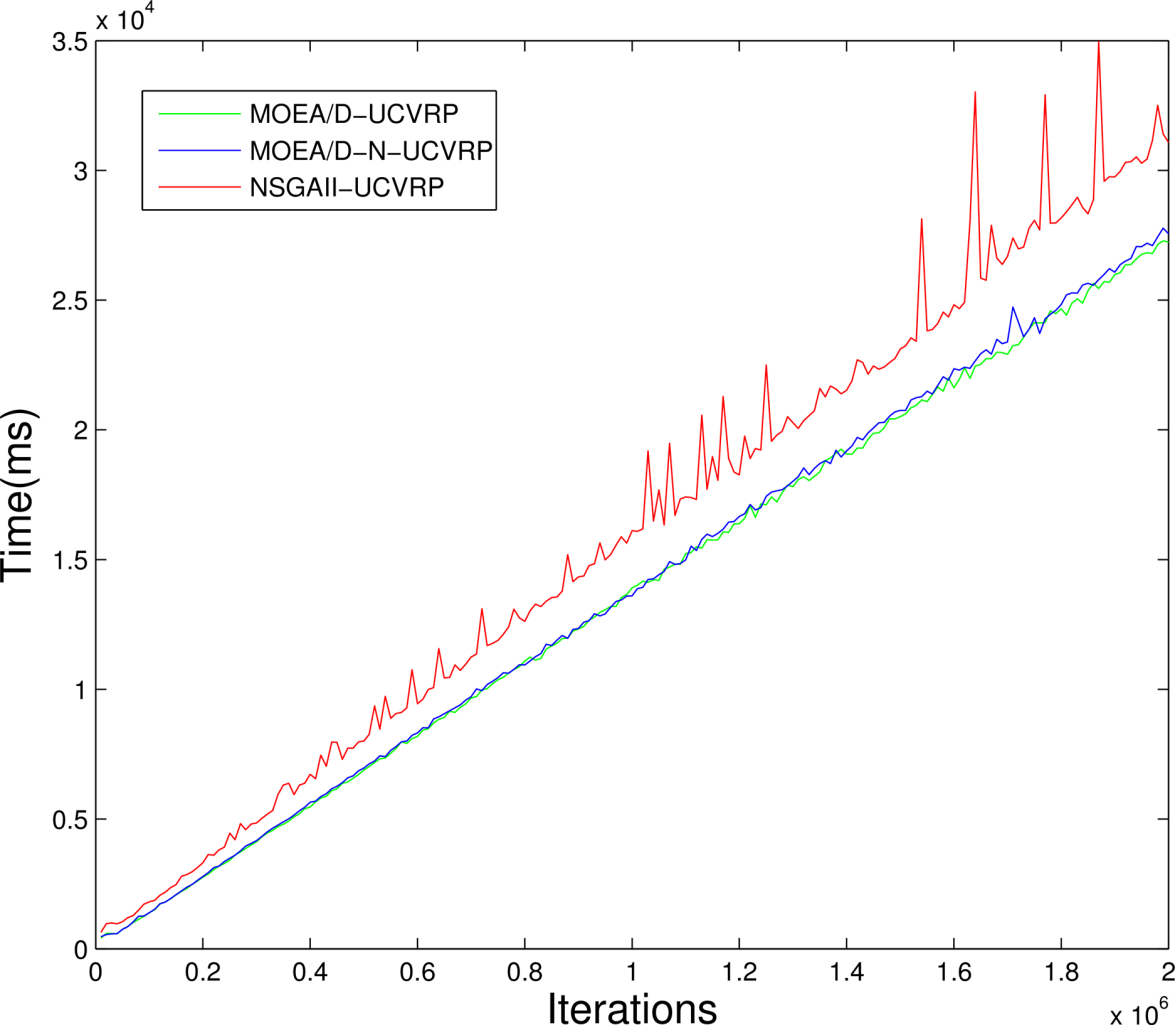
Relations between number of iterations and time cost of MOEA/D-N-UCVRP, NSGAII- UCVRP and MOEA/D- UCVRP.

We also conduct an experiment about the iteration times and the time cost of these three algorithms. Fig 14 shows the relations between the number of iterations and execution time of MOEA/D-N-UCVRP, NSGAII-CUVRP and MOEA/D-UCVRP. In the 10000 to 20000 iterations, the time cost of three algorithms shows a linear increase, the fluctuation of the NSGAII- CUVRP’s time cost is relatively large while the other two are more stable. Also, the time cost of MOEA/D-N- UCVRP is very close to MOEA/D- UCVRP.

## Conclusion

In this paper, the blood supply in emergency situation is introduced into the traditional Capacitated Vehicle Routing Problem, and a new multi-objective optimization problem is proposed according to the two factors of distance and weight. Aiming to achieve a real-life blood supply logistics, we study the blood’s temperature change due to the external environment, the heating agent (refrigerant) and time factor during transit, and propose an optimal method for calculating the mixing proportion between blood and appendage in different circumstance. Algorithmically, we use the combination of decomposition-based multi-objective evolutionary algorithm and local search method, in order to conduct a series of experiments on the VRP public dataset, which is included in S1 File. By comparing our technique with the traditional ones, we proved that our algorithm can obtain better optimization results and time performance. Our model can also be used in the food warming and route planning of food delivery, the transportation of frozen seafood and etc via UAVs.

## Supporting Information

S1 File VRP public dataset. (ZIP)
